# Alteration in cartilage matrix stiffness as an indicator and modulator of osteoarthritis

**DOI:** 10.1042/BSR20231730

**Published:** 2024-01-12

**Authors:** Jing Song, Xuemin Zeng, Chenzhi Li, Hongyan Yin, Sui Mao, Dapeng Ren

**Affiliations:** 1 The Affiliated Hospital of Qingdao University, Department of Stomatology Medical Center, Qingdao University, Qingdao, Shandong, CN, China; 2 Qingdao University Affiliated Qingdao Women and Children’s Hospital, Department of Stomatology Medical Center, Qingdao University, Qingdao, Shandong, CN, China; 3 Institute of Hybrid Materials, College of Materials Science and Engineering, Qingdao University, Qingdao, Shandong, CN, China

**Keywords:** cartilage, matrix stiffness, osteoarthritis

## Abstract

Osteoarthritis (OA) is characterized by cartilage degeneration and destruction, leading to joint ankylosis and disability. The major challenge in diagnosing OA at early stage is not only lack of clinical symptoms but also the insufficient histological and immunohistochemical signs. Alteration in cartilage stiffness during OA progression, especially at OA initiation, has been confirmed by growing evidences. Moreover, the stiffness of cartilage extracellular matrix (ECM), pericellular matrix (PCM) and chondrocytes during OA development are dynamically changed in unique and distinct fashions, revealing possibly inconsistent conclusions when detecting cartilage matrix stiffness at different locations and scales. In addition, it will be discussed regarding the mechanisms through which OA-related cartilage degenerations exhibit stiffened or softened matrix, highlighting some critical events that generally incurred to cartilage stiffness alteration, as well as some typical molecules that participated in constituting the mechanical properties of cartilage. Finally, *in vitro* culturing chondrocytes in various stiffness-tunable scaffolds provided a reliable method to explore the matrix stiffness-dependent modulation of chondrocyte metabolism, which offers valuable information on optimizing implant scaffolds to maximally promote cartilage repair and regeneration during OA. Overall, this review systematically and comprehensively elucidated the current progresses in the relationship between cartilage stiffness alteration and OA progression. We hope that deeper attention and understanding in this researching field will not only develop more innovative methods in OA early detection and diagnose but also provide promising ideas in OA therapy and prognosis.

## Introduction

Osteoarthritis is a whole joint disease characterized by progressive degradation of the articular cartilage as well as associated changes such as meniscal degeneration, subchondral bone remodeling, inflammation and fibrosis of synovium and surrounding joint tissues [1–4]. The major hallmark of osteoarthritis (OA) is destruction of the articular cartilage. Due to poor regenerative capacity of cartilage, there is almost no clinically effective method to reverse the loss of cartilage in OA [5]. One difficulty in dealing with the disease is that the clinical symptoms of OA appear only when it has become quite advanced [6]. Therefore, establishment of critical early events of OA that occur when the disease progression is still potentially reversible offers a promising researching field in OA diagnose, therapy and prognosis.

Cartilage matrix consists mainly of collagens, proteoglycans (PGs) and water, which give rise to mechanical properties of cartilage [7]. Alterations in cartilage mechanical properties, such as Young’s modulus or stiffness, have been reported in early stage of OA when no macroscopical or histological signs of cartilage destruction could be detected [8]. Clinically, *in situ* quantitative evaluation of articular cartilage stiffness during arthroscopy provides valuable information on identifying the early stage of OA. Various indentation technologies have been developed for deciphering early changes in mechanical properties of the cartilage in a clinical arthroscopic setup (for detail see review [9]). With the help of these techniques, there were growing evidences pointing out the relationship between cartilage stiffness and OA progression.

This review first provides a synopsis of recent advances in characterizing the details of altered cartilage stiffness during OA progression. Second, we aimed to summarize the potential molecular mechanisms underlying cartilage degeneration-related stiffening or softening of cartilage, as well as speculating how does the changed mechanical properties of cartilage affect the balanced anabolism and catabolism of chondrocytes embedded in extracellular matrix (ECM). Finally, the recent progress of using biomaterial stiffness as a cue for modulating chondrocyte function and phenotype will be discussed, depicting the promising design cornerstone of novel chondrocyte-instructive biomaterials for cartilage regeneration and repair during OA initiation.

## Cartilage stiffness as a sensitive marker of OA initiation

In order to uncover the stiffness of cartilage as a sensitive marker of OA initiation, several research groups measured the cartilage stiffness in various experimental setups mimicking early stage of OA. Using histology-based OA scoring systems such as OARSI (Osteoarthritis Research Society International) and ICRS (International Cartilage Repair Society), early alterations in Young’s modulus of cartilage were detected from patients with lower OA scores [10–12]. However, opposite results were obtained from these studies, which demonstrated either cartilage stiffening (from 320 to 335 MPa [11]) or cartilage softening (from 7 to 2 MPa [10], from 0.6 to 0.3 MPa [12]) as OA histological scores increased. It should be noted that as OA initiates, the gross stiffness of cartilage measured at larger scales was reduced, while the stiffness of more subtle structures (such as collagen fibers) of cartilage measured at nanoscale was increased. This conclusion could be reinforced by the other human studies which showed either decreased cartilage stiffness at the macroscopic level (from 2.1 to 0.13 GPa [13]) or increased collagen fiber stiffness at the nanometer scale (from 2.65 to 3.11 GPa [14]) during OA progression.

The spatial patterns of chondrocytes act as another biomarker for OA: chondrocytes rearrangement can be observed from single strings to double strings, followed by small clusters, and finally big clusters following OA progression [15]. Using this image-based OA stage defining system to characterize OA grade, the stiffness of cartilage correlated well with human OA progression, with single string pattern showing the highest modulus (∼200 kPa) while big cluster pattern showing the lowest modulus (∼25 kPa) [16–18].

In addition to the cartilage samples from OA patients, altered cartilage stiffness was also noticed in various animal OA models, such as post-traumatic osteoarthritis (PTOA) induced by destabilization of the medial meniscus (DMM), meniscectomy and anterior cruciate ligament transection (ACLT), as well as monosodium-iodoacetate (MIA) injection-induced OA [19–22]. Consistent with human samples, cartilage stiffness was altered shortly after constructions of these animal models when histological signs of OA were still not apparent. Overall, these studies demonstrated that the stiffness of cartilage is highly sensitive to the early-stage degradation of cartilage during OA progression in both clinical OA patients and experimental OA animal models.

## OA-related spatial variations in cartilage stiffness

Normally, the cartilage exhibits depth-dependent increase in the Young’s modulus from surface to deeper layers adjacent to subchondral bone [23]. The stiffness of cartilage surface generally declined as a result of OA development, e.g., from 25 to 3.4 kpa in aging-related human OA cartilage, from 1.5 to 0.4 MPa in ACLT-induced animal OA cartilage, from 1.95 to 1.6 MPa in talc injection-indued animal OA cartilage, as well as from 0.9 to 0.1 MPa in other established human OA cartilage [24–27]. However, this trend is in contradiction with other human studies which displayed elevated stiffness in both superficial and deeper zones of OA cartilage [14,28]. In addition, the surface of cartilage might suffer uneven destructions during OA progression, as revealed either by microscopic observation or histological staining, and the stiffness of cartilage surface correspondingly exhibited regional differences among distinctly destructed areas. The severely damaged zone of human cartilage surface was reported to be stiffer than normal human cartilage zone in one study (190.5 kPa in lesion zone and 34.86 kpa in healthy zone) [29] but softer than normal cartilage zone in another study (150 kPa in lesion zone and 1637 kpa in healthy zone) [30]. These disparate observations among different studies might be attributed to the distinct experimental factors, including the detecting area in cartilage, cartilage sample collection and preparation procedures, and the device through which cartilage stiffness was detected.

## Stiffness of cartilage ECM, PCM and chondrocytes in OA

Within the cartilage ECM, there is a narrow matrix region encompassing chondrocytes. This unique region, which compositionally and structurally differs from surrounding ECM, is approximately 2 to 4 μm thick and is called the ‘pericellular matrix’ (PCM). A sharp difference exists among the stiffness of cartilage ECM (∼100 kPa), PCM (20–40 kPa) and chondrocytes (0.5–1 kPa) from different species, such as human, porcine and murine [31,32]. The stiffness gradient from ECM and PCM to chondrocytes reflects the role of PCM as a transducer of mechanical signals from the ECM to the chondrocyte during joint loading [33].

In addition to their stiffness gradient, the stiffness of ECM, PCM and chondrocytes exhibit distinct zonal variance from cartilage surface to deeper zones [34–36]. PCM has the remarkable uniformity of Young’s modulus among the superficial (68 ± 5 kPa), middle (56 ± 4 kPa) and deep zones (58 ± 6 kPa) of cartilage [34]. In contrast, the Young’s modulus of both ECM and chondrocytes is depth-dependent, with the superficial zone of ECM (110–150 kPa) and chondrocytes (1.2–1.6 kPa) having greater stiffness than deep zone of ECM (∼100 kPa) and chondrocytes (0.69–0.78 kPa) [35,36].

The research team of Guilak manifested that PCM stiffness diminished significantly from human OA cartilage comparing with human normal cartilage, either by measuring the Young's modulus of PCM from isolated chondrons through micropipette aspiration technique (normal: 38.7 ± 16.2 kPa vs. OA: 23.5 ± 12.9 kPa), or by directly measure PCM stiffness in situ through atomic force microscopy (AFM) system (normal: 137 ± 22 kPa vs. OA: 96 ± 16 kPa) [37,38]. Another team of Hofmann provided consistent conclusions that PCM from human OA cartilage was softer than from human normal cartilage [16,39]. Furthermore, they displayed that PCM stiffness was correlated with the spatial arrangement of chondrocytes, with single strings showing the highest PCM stiffness (37.6 ± 5.4 kPa) and big clusters showing the lowest PCM stiffness (5.6 ± 0.8 kPa). Similarly, the Young’s modulus of ECM also declined in cartilage suffering OA (normal: 491 ± 112 kPa vs. OA: 270 ± 76 kPa) [38,39]. Notably, the extents to which ECM and PCM stiffness reduced during OA were comparable with each other, resulting in relatively stable ECM/PCM stiffness ratio in different stage of OA development [39]. Intriguingly, when comparing the sequential variation of PCM and ECM stiffness, Chery et al. [20] revealed that reduction in PCM stiffness occurred as early as 3 days after the onset of DMM-induced animal OA model, while decrement of ECM stiffness was only detectable after 1 week of OA initiation. This conclusion illustrated that cartilage PCM stiffness is a more sensitive marker than ECM stiffness, pointing out the critical role of PCM in driving OA initiation.

It is well known that chondrocyte behaves as a viscoelastic solid, the stiffness of which changes in response to altered stiffness of PCM or ECM [40]. For example, genetic or pharmacological modification of mice cartilage matrix led to alteration in chondrocyte stiffness in a similar trend [41]. Cytoskeleton elements, such as actin microfilaments, intermediate filaments, and microtubules, are important supporters of cellular structure and stiffness of chondrocytes. Using synthetic chemicals to specifically inhibit actin microfilaments, intermediate filaments and microtubules of cytoskeleton systems, studies have confirmed that both actin microfilaments and intermediate filaments contributed significantly to chondrocytes stiffness [42,43]. However, the extent to which actin microfilaments and intermediate filaments support chondrocytes stiffness seemed to differ among chondrocytes localizing in different zones of cartilage [42].

So far, the effects of OA on Young’s modulus of chondrocytes have not reached an agreement. Guilak’s group testified that *in vitro* chondrocytes isolated from OA patient samples had remarkably higher stiffness (0.5 kPa) than from normal samples (0.36 kPa) [44,45]. The authors attributed these contradictory results to different culturing time before micropipette aspiration test. In contrast, several other groups provided evidences that Young's modulus of chondrocytes from human or animal OA cartilage substantially declined as compared with chondrocytes from normal cartilage, through using *in vitro* micropipette aspiration testing (normal: 0.98 ± 0.14 kPa vs. OA: 0.68 ± 0.27 kPa), *in situ* AFM testing (normal: 0.096 ± 0.009 N/m vs. OA: 0.0347 ± 0.005 N/m) and *in vitro* mechanical compression testing (normal: 8.9 kPa vs. OA: 4 kPa), respectively [42,46,47]. Notably, these seemingly inconsistent results should be examined objectively, considering various unknown factors contributing to the stiffness of chondrocytes in different studies. Experimental factors such as specie, age, OA degree of cartilage donor from which chondrocytes were isolated, as well as the devices and methods through which stiffness of chondrocytes were measured, all affect the Young’s modulus of chondrocytes.

There are multiple mechanisms through which chondrocytes stiffness are altered during OA progression. First, disturbed expressions of chondrocyte cytoskeletons (F-actin or α-tubulin microtubules) by cellular OA inducers such as interleukin-1β (IL-1β), tumor necrosis factor-α (TNF-α), and sodium nitroprusside (SNP) contributed primarily to either increased or decreased stiffness of chondrocytes *in vitro* [48,49]. Second, pharmacal matrix degradation in ECM or PCM of ex vivo cultured cartilage leading to softened chondrocyte microenvironment resulted in corresponding chondrocyte stiffness alterations [43,50]. Third, extracellular calcium ion concentrations in cartilage are changed during OA, and *in vitro* cultured chondrocytes were reported to exhibit high stiffness in response to hyper-osmotic external environment, while hypo-osmotic pressure reduced Young’s modulus of chondrocytes [51,52].

Altogether, even though cartilage stiffness alteration acts as a sensitive marker of OA initiation, some details have not reached an agreement, such as whether cartilage stiffen or soften during OA development, how cartilage stiffness changed in relation to different layers of cartilage during OA development, and what parts do ECM, PCM and embedded chondrocytes played in determining the overall stiffness of cartilage during OA development. The inconsistencies among different studies need to be examined more carefully and objectively considering various experimental conditions that might affect the stiffness data (Table 1).

**Table 1:** Alteration in cartilage matrix stiffness during osteoarthritis progression

Species	Location	Joint	Cartilage sample	Major findings	Device	Scale	Ref.
Human	Cartilage surface	Knee	Established OA samples	Cartilage soften as OA progress (shear modulus)	Indentation probe	Millimeter	[8]
Human	Chondrons isolated from cartilage slices	Femoral head	Established OA samples	Cartilage PCM soften in OA (Young’s modulus from 38.7 ± 16.2 kPa to 23.5 ± 12.9 kPa)	Micropipette aspiration	Micrometer	[37]
Human	Cartilage surface	Knee	Established OA samples	Cartilage soften as OA progress (Young’s modulus from 0.5 ± 0.14 to 0.28 ± 0.12 MPa)	High-precision material testing device	Micrometer	[12]
Human	Cartilage surface	Knee	Established OA samples	Cartilage soften as OA progress (Young’s modulus and dynamic modulus)	High-precision material testing device	Micrometer	[57]
Dog	Cartilage surface	Femoral Head	Unilateral cranial cruciate ligament transection-induced OA models	Cartilage soften as OA progress (dynamic modulus from 1.5 ± 1.0 to 0.4 ± 0.3 MPa in the suffice layer; from 2.6 ± 1.5 to 0.6 ± 0.2 MPa in the deeper layer; from 4.3 ± 2.4 to 1.2 ± 0.4 MPa in the deepest layer)	Indentation-type AFM (IT AFM)	Micrometer	[25]
Human	Cartilage slices	Knee	Established OA samples	Collagen fibrils stiffen as OA progress (Young’s modulus from 2.26 to 3.94 GPa in the suffice layer; from 2.49 to 4.0 GPa in the deeper layer; from 2.63 to 4.09 GPa in the deepest layer)	Indentation-type AFM (IT AFM)	Nanometer	[14]
Human	Cartilage slices	Knee	Established OA samples	Cartilage PCM and ECM soften in OA (PCM Young’s modulus from 137 ± 22 to 96 ± 16 kPa; ECM Young’s modulus from 491 ± 112 to 270 ± 76 kPa)	Indentation-type AFM (IT AFM)	Micrometer	[38]
Rat/Human	Cartilage surface	Knee	Monosodium iodoacetate (MIA) and anterior cruciate ligament transection (ACLT)-induced OA models in rat; established OA samples in human	Cartilage soften as OA progress (stiffness from 118.43 ± 25.2 to 24.2 ± 15.3 MPa in MIA and 25.3 ± 4.8 MPa in ACLT; stiffness from 1.1 ± 0.5 to 0.5 ± 0.0 MPa in human)	Microindenters	Micrometer	[21]
Mice	Cartilage surface	Knee	Destabilization of the medial meniscus (DMM)-induced OA models	cartilage surface soften in early OA (indentation modulus reduced to 49%, 34% and 20% after 1,2 and 8 weeks of DMM),stiffen in late OA (indentation modulus elevatd to 82% after 12 weeks of DMM)	Indentation-type AFM (IT AFM)	Micrometer	[19]
Human	Cartilage surface	Knee	Established OA samples	Cartilage soften as OA progress (Young’s modulus from 5.7 ± 1.3 to 3.0 ± 2.2 MPa in anterior condyle, from 6.4 ± 2.2 to 2.5 ± 2.4 MPa in central condyle, from 8.0 ± 1.5 to 6.8 MPa in posterior condyle)	Indentation probe	Millimeter	[10]
Human	Cartilage surface	Knee	Established OA samples	Cartilage surface soften as OA progress (shear storage modulus from 0.9 to 0.1 MPa)	Indentation probe	Millimeter	[27]
Human	Cartilage surface	Knee	Established OA samples	Cartilage PCM and ECM soften as OA progress (Young’s modulus of ECM from 132 to 18 kPa; Young’s modulus of PCM from 65 to 13 kPa)	Indentation-type AFM (IT AFM)	Micrometer	[39]
Human	Cartilage surface	Knee	Established OA samples	Cartilage PCM soften as OA progress (Young’s modulus from 49.479 ± 5.395 kPa to 7.615 ± 0.788 kPa)	Indentation-type AFM (IT AFM)	Micrometer	[16]
Mice	Cartilage slices	Knee	Destabilization of the medial meniscus (DMM)-induced OA models	Cartilage PCM soften as OA progress (indentation modulus from 0.91 ± 0.09 MPa to 0.13 ± 0.04 MPa)	Indentation-type AFM (IT AFM)	Micrometer	[20]
Rabbit	Cartilage surface	Femoral head	Injections of saline and 10% surgical talc to induce OA; injection of platelet-rich plasma (PRP) to relieve OA	Cartilage soften in talc-induced OA (Young’s modulus from 1.95 ± 0.035 MPa to 1.60 ± 0.027 MPa), relieved by PRP (Young’s modulus 1.71 ± 0.022 MPa)	Indentation-type AFM (IT AFM)	Nanometer	[26]
Human mice	Cartilage surface	Knee	Established OA human samples; Destabilization of the medial meniscus (DMM)-induced OA models	Cartilage stiffen as OA progress (stiffness from 62.98 to 383.9 kPa)	Piuma nano-indentation	Nanometer	[29]
Human	Cartilage slices	Knee	Established OA samples	Collagen fibrils stiffen as OA progress (Young’s modulus)	Indentation-type AFM (IT AFM)	Nanometer	[11]
Human	Cartilage surface	Knee	Established OA samples	Cartilage stiffen as OA progress (Young’s modulus)	Indentation-type AFM (IT AFM)	Micrometer	[18]
Human	Cartilage surface	Femoral head	Established OA samples	Cartilage soften as OA progress (Young’s modulus from 349 ± 23 kPa to 283 ± 30 kPa)	Indentation-type AFM (IT AFM)	Micrometer and nanometer	[17]
Human	Cartilage surface	Knee	Established OA samples	Cartilage lesion zone is softer than remote zone (Young’s modulus from 1637 ± 138 kPa to 150 ± 50 kPa)	Nanoindenter probe	Micrometer	[30]
Human	Cartilage slices	Knee	Established OA samples (mild, moderate and severe)	Cartilage stiffen as OA progress (Young’s modulus)	Indentation-type AFM (IT AFM)	Nanometer	[28]

## Molecular mechanisms underlying cartilage degeneration-related stiffness alteration during OA

### Collagen and proteoglycan content

Cartilage matrix mainly comprised of collagens and PGs, both of which exert important biomechanical and structural roles in the cartilage. Collagens form the fibrillar network, providing cartilage with high tensile stiffness, while PGs interspersed within the collagen meshwork, creating the compressive stiffness due to their ability to attract water [53,54]. Therefore, collagens and PGs in cartilage produced non-overlapping modulus of cartilage, which resulted in a bimodal nanostiffness distribution, with PGs giving rise to the lower nanostiffness peak and collagen fibrils yielding the higher nanostiffness peak [55]. Broad-spectrum enzymatic digestion of cartilage with elastase was able to attenuate buck stiffness of cartilage at microscale, while specific digestion of the PGs moiety by cathepsin D had little effect on microstiffness but reduced nanostiffness of cartilage [56].

During OA development, the decreased content of collagens and PGs were parallel with the reduction in cartilage stiffness [57]. Using *in vitro* enzymatic digestion of cartilage explant to mimic cartilage matrix degradation in OA, Lewis et al. confirmed that cartilage stiffness declined in response to collagenase digestion in a concentration-dependent manner (10 μg/ml collagenase: 7.27 ± 0.03 MPa, 1 mg/ml collagenase: 5.3 ± 0.13 MPa, 5 mg/ml collagenase: 2.96 ± 0.2 MPa) [58]. Furthermore, the spatial inhomogeneities of stiffness between cartilage ECM and PCM could also be explained by their distinct abundance of collagens and PGs [59]. Overall, the decrement of collagens and PGs in cartilage contributes to OA-related decrease in cartilage stiffness.

### Collagen fiber orientation

In addition to cartilage matrix content, the alignment of collagen fibers is also a determinate factor of compressive modulus of cartilage. Collagen fibrils normally organized in a mesh-like pattern parallel to the surface of cartilage in the superficial layers, gradually become randomly aligned in the intermediate layer, and oriented perpendicular to the subchondral bone in the deep layer [13,23,60]. This pattern correlated well with the stiffness gradient from articular surface to deep layer. Thus, perpendicularly oriented collagen fibrils that are endowed with higher Young’s modulus are resistant against mechanical loading. Accordingly, mature or adult cartilage was reported to be stiffer than immature or adolescent cartilage, which is consistent with the multidirectional organization of collagen fibrils in immature cartilage and more perpendicularly aligned collagen fibrils in mature cartilage [61,62].

With regard to OA, several studies reported the collagen fibril reorientation during OA development. Makela et al. [63] found that collagen fibril orientation shifted from the parallel towards the perpendicular pattern in human hip OA samples, and this change impaired collagen network and the stiffness of cartilage. Another study reported that dispersion of collagen fibers alignment angle increased with OA progression in both the superficial and deep layers of human knee OA cartilages, while the primary orientation of collagen did not change in contrast with healthy counterparts [64]. By using ACLT-induced animal model, several groups investigated the collagen fiber orientation in ACLT-induced OA cartilage. There were minor changes in collagen orientation angle 2-week post ACLT surgery, but collagen reorientated perpendicularly to cartilage surface 4-week post ACLT comparing with collagen that run parallel to cartilage surface in normal group [65–67]. In cartilage samples from 9-week post ACLT group, the collagen orientation angle increased in the superficial layer but decreased for the rest of cartilage [68]. Even though these studies demonstrated altered collagen fibril arrangement during OA development, the correlation between altered cartilage stiffness and collagen fibril organization was still inconclusive. Recently, Inamdar et al. [69] performed an *ex vivo* cartilage explant model treated with IL-1β to simulate the inflammatory condition in OA. Their data uncovered that collagen fibrils in normal cartilage explant reversibly change the width of orientation distribution under mechanical loading, while maintaining a consistent average orientation. In IL-1β treated cartilage explant, the collagen rearrangement under cyclic loading is disrupted, which was associated with reduced cartilage stiffness [69]. Based on these studies, collagen fiber reorientation was supposed to be related with alteration of cartilage stiffness in both physiological and pathological conditions, even though the direct evidence for this conclusion is still lacking.

### Structural alterations of cartilage matrix

During the initial stage of cartilage degeneration, structural changes of cartilage matrix (e.g., fibrillation of the collagen fibrils, disruption of the collagen network, decreased proteoglycan aggregation) occur, preceding the degradation of matrix components. The reduced interconnections between cartilage matrix components resulted in decreased gross stiffness of cartilage [8,24]. However, when measuring the stiffness at the nanoscale, it should be noted that loss of tightly interconnected tropocollagen was accompanied with generation of more aligned collagen bundles, which give rise to some stiffer collagen fibrils during early stage of OA [11]. In general, the disintegrated collagen network accounted for the reduction in gross stiffness of cartilage during OA initiation, while the incidental formation of aligned collagen bundles in the process of cartilage structural disruption brought about increased Young’s modulus.

Aging-related OA is characterized by stiffened cartilage matrix, which results from the accumulation of advanced glycation end products (AGEs) [70]. AGEs are regarded as a major factor in driving nonenzymatic collagen cross-linking in cartilage matrix, thereby increasing cartilage stiffness [71]. This conclusion was further confirmed using *ex vivo* cartilage culture in the presence of threose or lysine, which either promoted or inhibited AGEs levels and consequently increased or reduced cartilage stiffness [72]. Notably, the generation of AGEs in cartilage during aging makes collagen network more brittle, leading to loss of elasticity. This subsequently increased susceptibility of the cartilage collagen network to mechanical failure during joint activity, thus predisposing aging cartilage to OA development.

### Involvement of specific cartilage matrix molecule

**Figure 1:**
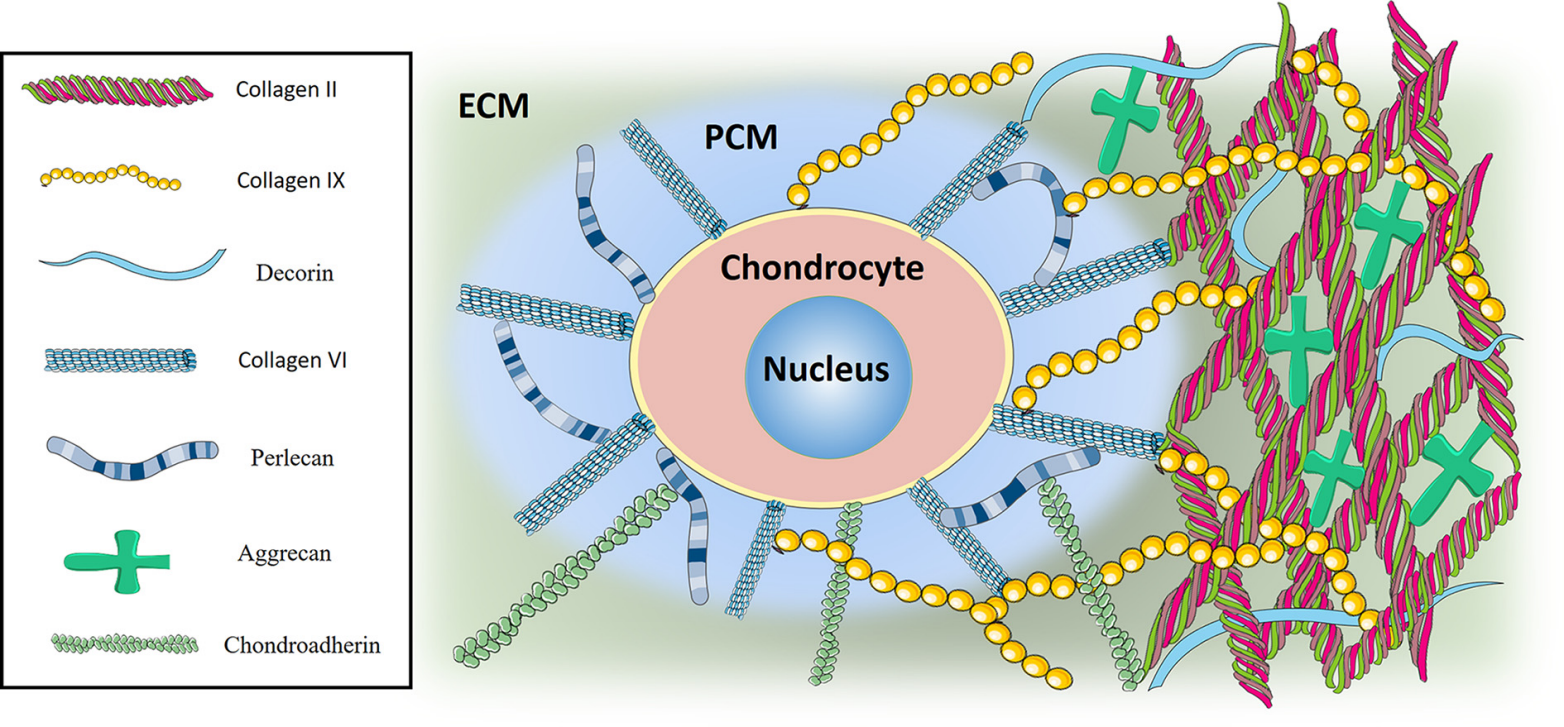
Matrix molecules constituting cartilage matrix stiffness Major matrix molecules in cartilage matrix that has been reported to be related with cartilage stiffness. Some of these, such as collagen VI and perlecan, were found exclusively in cartilage PCM, while other molecules like collagen II, collagen IX, aggrecan, decorin and chondroadherin were found predominately in cartilage ECM. These molecules covalently bond with each other, which together constitute cartilage matrix stiffness.

Up to date, several specific collagens and PGs were reported to contribute to the inherent stiffness of cartilage, and the depletion of these molecules are associated with altered cartilage stiffness and consequently with OA progression. In this part of review, we listed some of these typical matrix molecules that has been reported (Figure 1).

### Collagen VI

Collagen VI is heterotrimeric protein playing a bridging role in connective tissues, where it forms a flexible network interlinking collagen I, II, IV, PGs, hyaluronan, and cells. In articular cartilage, collagen VI is exclusively localized to the pericellular matrix (PCM). Notably, the biomechanical properties of cartilage PCM is determined by the presence of collagen VI, which serves as a defining factor contributing to the lower elastic modulus of PCM in contrast with ECM that contains no collagen VI [34]. In cartilage samples from OA patients, the progressive reduction in PCM stiffness from regions with minor destruction to regions with severe destruction correlated well with gradual loss of type VI collagen, further demonstrating the primary role of collagen VI in maintaining cartilage PCM stiffness [16]. Mutated mice with targeted gene disruption of collagen type VI α 1 chain (*Col6a1*) in cartilage also provided strong evidences that collagen VI is a major factor constituting the elastic modulus of cartilage PCM [73,74]. However, these two studies proposed opposite conclusions about the effect of *Col6a1* silencing on aging-related OA. The study confirming that aging accelerated cartilage degeneration in *Col6a1*-/- mice focused on the hip joint (PCM from wide-type mice: 300 kpa, PCM from *Col6a1*-/- mice: 80 kPa) [73], while the other study identifying delayed OA progression in Col6a1-/- mice collected samples from knee joints (PCM from wide-type mice: 58Kpa, PCM from *Col6a1*-/- mice: 46 kPa) [74]. As joint mobility is a predisposing factor in the development of osteoarthritis, the inconsistent conclusions from these two studies might resulted from different joints with distinct mobilities.

### Collagen IX

Studies have shown that mice deficient in *collagen IX* developed osteoarthritis-like changes in their knee joints [75]. Collagen IX is covalently bound to the collagen II fibril and mediates interactions between the collagen fibrils and other components of the extracellular matrix. Lack of collagen IX may alter the molecular architecture and thus the mechanical properties (e.g., stiffness) of the articular cartilage. However, the effect of collagen IX deficiency on cartilage stiffness was only detected in aged mice [24]. This might point out potential interactions between AGEs and collagen IX in determining the Young’s modulus of cartilage during aging.

### Aggrecan

Aggrecan is the most abundant PG of the cartilage matrix. The protein contains the chondroitin sulphate (CS) chains with high negative charge density, being able to attract counter ions and drawing water molecules into the tissue, thus creating a positive osmotic pressure which enables articular cartilage to resist compressive forces. *Ex vivo* culture of cartilage with chondroitinase-ABC or ADAMTS-4 (A Disintegrin and Metalloproteinase with Thrombospondin motifs-4), which cleaves the CS chains or core protein of aggrecan, resulted in decreased modulus of cartilage, suggesting an important role of aggrecan in rendering compressive resistance of cartilage matrix [59,76].

Since aggrecan is enzymatically degraded during OA progression, the observed cartilage softening in OA was possibly associated with aggrecan proteolysis. This conjecture was confirmed in two different animal OA models. One study applied PTOA model, in which the authors proved that production of VDIPEN341, the cleaved neoepitope of aggrecan, was increased in PTOA animals. Furthermore, the reduced cartilage stiffness in PTOA was partially counteracted by injection with GM6001, which is a specific inhibitor of aggrecan proteolysis [19]. The other study developed an aging joint overuse model to generate cartilage degeneration. They observed that joint overuse provoked cartilage to soften in wild-type mice, but cartilage softening was mitigated in aggrecanase-resistant mutated mice, implying the critical function of aggrecan in maintaining mechanical properties of cartilage in response to joint activities [77].

The negatively charged aggrecan anchored to hyaluronic acid (HA) within the matrix of articular cartilage. Using anionic nanoparticles conjugated with the HA-binding peptide as a substitute of aggrecan, Deloney et al. illustrated that treating aggrecan-depleted cartilage explants with the nanoparticles restored the cartilage compressive stiffness to normal levels, highlighting the prospective of targeting aggrecan to prevent cartilage degeneration in PTOA [78].

Accordingly, all these studies agreed that degraded aggrecan in OA accounted for the reduction in cartilage stiffness. Ultimately, the definitive role of aggrecan on cartilage stiffness was convinced by Alberton et al. with use of aggrecan knock-out mice [79]. Interestingly, their findings indicated that genetic depletion of aggrecan incurred to increased Young’s modulus of cartilage (wide-type: bimodal nanostiffness of 285.47 kPa and 484.61 kPa, aggrecan-/-: bimodal nanostiffness of 485.95 and 800.04 kPa), and resulted in higher incidence of spontaneously aging-related OA. Currently, there is no reasonable explanation for these disparate effects of aggrecan on cartilage stiffness. Different animal models (induced-OA versus spontaneous OA) and aggrecan depletion methods (enzymatic digestion versus genetic silencing) should be rigorously considered in order to reach the conclusion.

### Perlecan

Perlecan, encoded by the heparan sulfate proteoglycan2 (*Hspg2*) gene, is found exclusively in cartilage PCM, particularly in the pericellular region adjacent to chondrocytes. With regard to the mechanical properties of cartilage, perlecan was proved to be another major factor contributing to lower stiffness of PCM than ECM in cartilage, in addition to collagen VI [80]. The exact mechanism by which perlecan contributes to lower elastic moduli is currently unknown, but could be due in part to the heparan sulfate (HS) chain of perlecan protein, since digestion of the HS chains lead to the stiffer PCM [80]. In addition to the function of perlecan on PCM modulus of cartilage, the buck ECM stiffness was also decreased in perlecan knockout mice [41]. The authors assumed that various matrix molecules, such as collagen II fibril, fibrillin-1, aggrecan, connect with perlecan in cartilage matrix. Disruption of perlecan led to disassembling of this network, thus attenuating the elastic modulus of cartilage [41]. In the context of OA, Danalache et al. provided evidences that the reduction in cartilage stiffness during OA progression was correlated with diffusing perlecan staining, further demonstrating that perlecan degradation as a result of OA initiation provoked cartilage matrix disorganization and softening [16].

### Chondroadherin

Chondroadherin (CHAD), a class IV small leucine rich proteoglycan (SLRP), is localized in both the in the pericellular and territorial matrix of cartilage and plays a critical role in regulating linkages between collagens and other matrix molecules. Batista et al illustrated that *chad* depletion resulted in softer cartilage matrix in the superficial area, while had no effect on the middle and deep layers of cartilage [81]. This is partially due to relatively low abundance of aggrecan in the superficial cartilage. However, the weakening of cartilage upon *chad* deletion did not provoke spontaneous OA, in contrast to development of OA phenotype in other SLRP-deficient mice models such as *biglycan*-null and *fibromodulin*-null mice [82,83]. These disparities are likely associated with the differences in the molecular structures of CHAD compared to other SLRPs, in which CHAD does not have a glycosaminoglycan (GAG) side chain like most other SLRPs do.

### Decorin

Decorin (DCN) is a member of the class I SLRPs that also participate in ECM assembly and mechanical properties in a way similar to CHAD. However, *dcn* deficient mice exhibited stiffer cartilage matrix (wide-type: bimodal nanostiffness of 117KPa and 324KPa, dcn-/-: bimodal nanostiffness of 284KPa and 399KPa), as opposed to *chad* deficient mice [84]. Gronau et al. extensively explored the mechanism through which *dcn* depletion increased the cartilage stiffness [84]. They proposed that DCN sequestered transforming growth factor β (TGFβ), and genetic depletion of *dcn* released TGFβ that activated carbohydrate sulfotransferase 11 (Chst11) expression, which induced an increased negative charge density of cartilage matrix, which attracted water and resulted in augmented compressive stiffness [84]. Furthermore, *dcn* deficient mice was resistant to exercise-induced OA in part due to the protective effect of TGFβ, in contrast with the aforementioned *biglycan*-null and *fibromodulin*-null mice that predisposed to spontaneous OA [82–84].

## Matrix stiffness as a promising microenvironmental factor in cartilage engineering

Matrix-assisted autologous chondrocyte implantation (MACI) has gained a lot attractions for its applications in repairing articular cartilage lesions during OA, and in optimizing biomimetic scaffold designs to promote ECM deposition by the implanted chondrocytes. Biochemical factors, such as hyaluronic acid and chondroitin sulfate, have frequently been incorporated into synthetic scaffolds to promote chondrocyte differentiation and cartilage matrix production. Another vital consideration in manufacturing scaffolds is its biomechanical parameters. Biomaterials with optimal mechanical properties should resemble that of cartilage, which is characterized by its non-linear elasticity, viscosity, permeability, compressibility, heterogeneity and anisotropy. A recent review by Petitjean et al. [85] elaborated these characters of native cartilage and pointed our future directions in developing cartilage-like biomaterials.

In contrast, the present review only focused on the biomaterials with controllable stiffness to emulate cartilage matrix stiffness under physiological and pathological conditions. Numerous studies have investigated the potential effects of scaffold stiffness on the cytoskeleton, metabolism, and proliferation of chondrocytes that were either seeded on top of scaffolds (2D *in vitro* culture) or embedded within scaffolds (3D *in vitro* culture). In this part of the review, we will list some of the recent findings of these researches and compare the consistent and inconsistent results (Table 2). In addition, the mechanisms of how microenvironmental stiffness regulate chondrocyte behaviors proposed by some of these studies will also be discussed.

**Table 2:** Scaffolds with tunable stiffness affect the chondrogenic phenotype of chondrocytes

Scaffold fabrication	Stiffness	Cell	2D/3D	Influences	Gene expression (low stiffness →high stiffness)	maintaining chondrogenic Phenotype	Ref.
Polyacrylamide (PA) gel with different concentrations of bis-acrylamide	4, 10, 40, 100 kPa	Porcine chondrocytes isolated from the condyle of 3- to 6-month-old pigs	2D	Proliferation; cytoskeleton organization	Col II, aggrecan ↓, Col I↑	Low modulus	[86]
Gelatin cross-linked with glutaraldehyde (GA)	100-150 MPa	Bovine chondrocytes isolated from 8-month-old calves	2D	Proliferation	Col II, aggrecan↑, Col I↓	High modulus	[87]
Poly(ethylene glycol) dimethacrylate (PEGDM) dissolved in chondrocyte medium at 10, 15 or 20 wt.%	60, 320, 590 kPa	Bovine chondrocytes isolated from the patellar femoral groove of 1- to 3-week-old calves	3D	sGAG and collagen deposition, matrix degradation products (C1,2C fragment,the N-terminal FFGV fragment)	Col II↓, Col VI, aggrecan↓, MMP-1↑, MMP-3, MMP-13↑	Low modulus	[99]
PA gel with different concentration of the cross-linker, piperazine diacrylamide	0.2, 0.5, 1.1 MPa	Murine chondroprogenitor cell line ATDC5; murine chondrocytes isolated from femoral condyles and tibial plateaus of newborn mice	2D	Proteoglycan deposition	Sox9, Col II, aggrecan	Opitmal modulus	[100]
PEGDM hydrogel	2–6 kpa	Chondrocytes isolated from the tibial plateaux and femoral condyles of OA patients	3D	Proliferation; cytoskeleton organization; phenotype (CD14/CD90); GAG and collagen deposition; MMP3 and MMP13 deposition	NA	Low modulus	[101]
PA gel with different concentrations of bis-acrylamide	1, 11, 90 kPa	Chondrocytes isolated from the knee joints of goats	2D	Morphology, cytoskeleton organization, cellular stiffness	Col II, aggrecan↓	Low modulus	[89]
3D collagen gel with ribose-mediated AGE accumulation or with LOX-conditioned medium; 2D polyacrylamide gels with with different concentrations of bis-acrylamidevariations	4–31 kPa (2D gel)	Murine chondrocytes isolated from femoral condyles and tibial plateaus of mice	2D/3D	Proteoglycan deposition, catabolism and anabolism, morphology, cytoskeleton organization	Sox9, Col II, aggrecan↑, MMP3, MMP13↓	High modulus	[70]
Gelatin hydrogels with different degree of photo-cross-linking	3.8, 17.1, 29.9 kPa	Bovine chondrocytes isolated from 9-week-old calves	3D	Morphology, cytoskeleton organization, proteoglycan deposition	Col II, aggrecan↑	High modulus	[98]
Polydimethylsiloxane (PDMS) via thermal cross-link with different proportions of monomer and initiator	1.4, 6, 16, 54, 135 kPa	Murine chondrocytes isolated from newborn rats	2D	Morphology, cytoskeleton orgnizationn and tension	Col II, aggrecan↓	Low modulus	[90]
PDMS via thermal cross-link with different proportions of monomer and initiator	2.21 MPa, 54.47, 2.13 kPa	Murine chondrocytes isolated from femoral condyles and tibial plateaus of newborn mice	2D	Morphology, cytoskeleton orgnization, mechanical parameters (elastic modulus, instantaneous modulus, relaxed modulus, viscosity)	NA	Low modulus	[91]
Chitosan-hyaluronic acid dialdehyde (CH-HDA) hydrogels with increasing concentration of HDA	130, 181, 199 kPa	Chondrocytes isolated from the knee joints of rabbits	3D	Morphology, viability, collagen II, proteoglycan deposition	Col II, sGAG↑	NA	[92]
Photo-cross-linked PEGDMA hydrogels with covalent incorporation of small molecule anionic or zwitterionic residues	5, 10, 15, 30 kPa	Murine chondrocytes isolated from newborn mice	3D	Proliferation, collagen II, proteoglycan deposition	Col II, sGAG↓	Low modulus	[88]
PDMS via thermal cross-link with different proportions of monomer and initiator	2.5, 1.36 MPa, 129.17, 27.42 kPa	Chondrocytes isolated from human normal joints with fracture	2D	Cytoskeleton orgnization	Sox9, Col II	Opitmal modulus	[29]
Gelatin scaffolds with two different concentrations of glutaraldehyde	20, 200 kPa	Chondrocytes isolated from the lesion and remote zones of joints from OA patients	3D	Col II and aggrecan deposition	Sox9, Col II, aggrecan, Col I, MMP13, TIPM1	High modulus	[30]
PDMS via thermal cross-link with different proportions of monomer and initiator	197, 78, 54, 2 kPa	Murine chondrocytes isolated from newborn mice	2D	Average cellular [Ca^2+^] response rates, oscillation peak [Ca^2+^] amplitude, frequency of [Ca^2+^] oscillations	TRPV4, Piezo1/2	NA	[106]
PA gel with different concentrations of bis-acrylamide	5, 50 kPa and 1 GPa	Murine chondroprogenitor cell line H5 clone	2D	Morphology, cytoskeleton orgnization, primary cilium (length, frequency)	NA	Low modulus	[93]
PDMS via thermal cross-link with different proportions of monomer and initiator	45, 450 kPa	Murine chondrocytes isolated from newborn mice	2D	Morphology, cilium (synapses) length and frequency, cytoskeleton orgnization	Laminin β1, focal adhesion protein FAK	Low modulus	[94]
Polyethylene glycol (PEG) hydrogels functionalized with RGD peptides was used as soft substrate, glass slides were used as the stiff substrate	0.8 kpa (soft)	Murine chondrocytes isolated from newborn Sprague Dawley rats	2D	Morphology, cytoskeleton orgnization	Sox9, Col II, aggrecan↓	Low modulus	[95]
Polyacrylic acid (PAA) hydrogel was used as soft substrate, plastic plates were used as the stiff substrate.	NA	Chondrocytes isolated from knee joints of OA patients	2D	Morphology, cytoskeleton orgnization	Sox9, Col II↓, Col I↑, IL-6, IL-10↓	Low modulus	[96]
Polyacrylamide (pAAm) gels and gelatin-based hydrogels (GelMA)	5, 21, 100 kpa (pAAm); 2,71 Kpa (GelMA)	Murine chondrocytes Isolated from young and aged mice	2D(pAAm); 3D(GelMA)	Morphology, cytoskeleton orgnization	Col II, aggrecan	Low modulus	[97]

### Effect of scaffold stiffness on chondrocyte proliferation

In 2D culturing on synthetic substrates such as polyacrylamide (PA) gels and electrospun cross-linked gelatin fibers, chondrocyte proliferation was demonstrated to increase as the substrates became stiffer [86,87]. In contrast, other studies displayed opposite effects of scaffold stiffness on chondrocytes embedded within agarose hydrogels or poly (ethylene glycol) dimethacrylate (PEGDMA) hydrogels [88]. These contrary results were partially attributed to different *in vitro* culturing methods (2D versus 3D) and different fabricated scaffolds. However, it is also possible that scaffold stiffness inhibited chondrocyte proliferation when the Young’s modulus was relatively low (e.g. below 50 kpa) [88], but promoted chondrocyte proliferation at higher Young’s modulus (e.g. from 100 kpa to 100 Mpa) [86,87].

### Effect of scaffold stiffness on chondrocyte morphology, cytoskeleton and mechanical properties

Chondrocytes are normally round in cartilage, but gradually adapt a polygonal morphology during dedifferentiation, which is common in traditional *in vitro* culture. In spite of various synthetic scaffolds with stiffness ranging from MPa to kPa, most researches agreed with the notion that chondrocytes tend to maintain their physiologically spherical morphology when cultured in softer materials compared with stiffer ones [70,89–97]. So far, only one study made the opposite conclusion that scaffold with higher stiffness better retained round chondrocytes than scaffold with lower stiffness [98]. The authors attributed this to differences of stiffness range and material properties between their study and others.

Chondrocyte morphology is tightly controlled by cytoskeletal fiber formation and organization. Through mechanosensing the stiffness of scaffolds, chondrocytes sensitively regulated their cytoskeleton in order to match themselves with the microenvironments. In consistent with changed morphology in response to different stiffness, chondrocytes actin filament systems consisting of F-actin and vinculin were found to be prominent and intensively organized when cultured with stiffer matrix but became diffused and disorganized in softer matrix [70,89–91,93–97].

The mechanical properties of chondrocytes, such as elastic modulus, stiffness and viscosity were all determined by the cytoskeletal tension. By applying AFM to measure these mechanical parameters of chondrocytes, studies have demonstrated that stiffer scaffolds enhanced both elastic modulus and viscoelastic parameters (instantaneous modulus, relaxed modulus and apparent viscosity) of chondrocytes [91]. In addition, cellular stiffness of chondrocytes was also reported to be elevated in gels with higher Young’s modulus [89]. Altogether, these *in vitro* studies manifested chondrocytes displayed altered mechanical properties within scaffolds of various stiffness in a way similar to the changed mechanical properties of chondrocytes in situ in cartilage samples with distinct matrix stiffness, which were discussed in the previous part of this review before.

### Effect of scaffold stiffness on chondrocyte phenotype and metabolism

Phenotypically, chondrocyte dedifferentiation involves decreased expressions of chondrogenic markers collagen II, aggrecan, SRY-box transcription factor 9 (Sox9) and increased expressions of fibroblastic markers collagen I and versican. Lots of methods, such as addition of growth factors, substrate coating, hypoxia and 3D culture, have been developed to maintain chondrocyte phenotype during *in vitro* culture. In parallel with chondrocyte morphology and cytoskeleton that displayed typically dedifferentiated character in stiffer microenvironments, the expressions of collagen II, aggrecan, SOX9 diminished while collagen I level elevated in chondrocytes, which suggested transition from chondrogenic to fibroblastic phenotype upon culture with stiffer scaffolds [86,88–90,95–97,99–101]. In addition to these chondrogenic and fibroblastic markers, the ratio of CD14 (a lipopolysaccharide receptor found on freshly isolated chondrocytes) to CD90 (a glycosylphosphatidylinositol-anchored glycoprotein associated with cellular proliferation) was also used to quantify chondrocyte phenotype maintenance, which was enhanced in softer environment but reduced in stiffer environment [101].

Jutila et al. [102] and McCutchen et al. [103] conducted high-performance mass spectrometry, and identified distinct metabolomic changes of chondrocytes in response to different matrix substrate stiffness. The stiffness of material scaffolds could affect not only the matrix proteins (proteoglycan, collagen II, collagen VI and aggrecan) deposition around chondrocytes but also secretions of matrix metalloproteinases (MMPs) and tissue inhibitor of metalloproteinase (TIMPs) by the chondrocytes. Generally, chondrocytes cultured in softer scaffolds that approach the physiological stiffness of cartilage preferred more matrix protein deposition and less MMPs secretion [86,88–91,95–97,99,101]. Moreover, scaffold stiffness was also reported to regulate inflammatory response of chondrocytes, with less pro-inflammatory factor interleukin-6 (IL-6) and more anti-inflammatory factor interleukin-10 (IL-10) released in softer scaffold [96]. However, there were still some studies providing opposite evidences that stiffer scaffolds tended to maintain chondrocytes in the anabolic state with higher expressions of matrix proteins and lower expressions of MMPs [30,70,87,98]. The range of stiffness should be considered to explain the inconsistency. In other words, there might be the optimal stiffness to some extent, which maximally promoted anabolism and inhibited catabolism of chondrocyte. Supporting this conjecture, two studies that either used polydimethysiloxane (PDMS) or polyacrylamide (PA) which covered stiffness ranging from 27.42 kPa to 2.67 MPa and from 1.1 to 0.2 MPa, demonstrated that the scaffold stiffness approaching the physiological stiffness of cartilage had a better effect on maintaining chondrocytes in the anabolic state than scaffolds with higher or lower stiffness [29,100]. In addition to the stiffness range, many other factors such as the fabricating materials, the culturing pattern (2D versus 3D), the sources of chondrocytes (primary chondrocytes versus chondroprogenitor cell line, healthy versus osteoarthritis donors, isolation from different joints), are all different in these studies, thus contributing to distinct regulations of anabolic and catabolic metabolisms.

### Molecular mechanisms of scaffold stiffness-dependent regulation of chondrocyte cytoskeleton and metabolism

The mechanisms of how microenvironmental stiffness regulates cytoskeleton is that cells first exert contraction forces onto the microenvironmental substrate and subsequently adjust their cell-ECM adhesion strength via the changes of focal adhesion plaques (FA), which finally result in the homeostasis between intracellular forces based on cytoskeletal contractility and extracellular forces coupling to the ECM stiffness [104]. FA serve as the core bridge between ECM component and cytoskeleton by anchoring to both the extracellular matrix proteins and the cytoskeletons. Zhou et al. [94] proposed that focal adhesion plaques kinase (FAK) had a direct bonding with laminin β1, an important extracellular matrix glycoprotein, in chondrocytes. Furthermore, short interfering RNA (siRNA) mediated reduction of FAK disturbed the cytoskeleton organization in stiffer microenvironment. Expressions of four potential mediators of cytoskeleton re-organization in chondrocytes, PDZ and LIM domain 3 (PDLIM3), endothelial cell surface expressed chemotaxis and apoptosis regulator (ECSCR), myosin light chain phosphorylatable fast skeletal muscle (MYLPF), and CD93 were increased in stiffer substrate, while expressions of other six mediators, keratin 16 (KRT16), myosin IA (MYO1A), growth associated protein 43 (GAP43), leiomodin 1 (LMOD1), serine/threonine kinase 33 (STK33) and proline rich 5 (PRR5), were increased in softer substrate. Altogether, the axis of laminin β1-FAK modulated its downstream cytoskeletal organization mediators, and contributed to chondrocyte cytoskeletal reorganization in response to different microenvironmental stiffness.

Ras homolog gene family member A (RhoA) is a small GTPase protein, and Rho-associated protein kinase (ROCK) is a downstream effector of RhoA. RhoA/ROCK pathway has been demonstrated to be the main regulator of cellular stress fiber formation and cytoskeletal reorganization. In addition, this pathway has also been confirmed to be related with OA progression by modulating chondrocyte metabolic activities. By culturing chondrocytes in polydimethylsiloxane (PDMS) materials with various stiffness, Zhang et al. [90] corroborated the positive correlation between RhoA/ROCK activation and PDMS stiffness, and the negative correlation between PDMS stiffness and chondrocyte anabolic gene expression, such as collagen II and aggrecan. Therefore, activation of RhoA/ROCK in chondrocytes embedded in stiffer substrate seemed to repress the anabolism of chondrocytes. In further support of this conclusion, inhibition of RhoA and ROCK both abolished stiffening-mediated up-regulation of matrix-degrading enzymes like MMP3 and MMP13, and down-regulation of collagen II and aggrecan [70]. Furthermore, phosphorylation of myosin light chain (MLC) mediated the RhoA/ROCK-regulation of anabolic or catabolic gene expression of chondrocytes [70]. Another potential downstream pathway of RhoA/ROCK in manipulating anabolic or catabolic responses of chondrocytes to matrix stiffness was TGFβ/SMAD3 [100]. Softer material not only promoted more TGFβ expression and autocrine in chondrocytes but also activated downstream SMAD3 phosphorylation and nuclear localization. This finally resulted in chondrogenic phenotype by expressing *Sox9*, *Collagen II* and *aggrecan*, and depositing PGs.

In addition to the RhoA/ROCK pathway that bridge the matrix stiffness and chondrocyte metabolism, yes-associated protein (YAP) was illustrated to be another player in mediating chondrocyte catabolism in stiffer microenvironment. This was associated with YAP dephosphorylation and subsequent nuclear translocation. Both *in vivo* and *in vitro* studies demonstrated that stiffer microenvironment in OA was parallel with more nuclear YAP protein and less cartilage matrix deposition [29,95]. Furthermore, genetic ablation of YAP or pharmacological inhibition of YAP nuclear translocation not only promoted chondrocyte anabolism in stiffer scaffold but also rescued cartilage degradation in animal OA models. Ras-related protein 2a (RAP2A) is a small G protein with GTP-enzyme activity that relays ECM rigidity signals into mechanosensitive cellular activities via YAP. The expression of RAP2A negatively corresponded with matrix stiffness and acted as an upstream factor of YAP activation via Hippo pathway [95]. Thus, these two studies together proposed that stiffer microenvironment induced loss of chondrogenic phenotype through reduced expression of RAP2A and subsequent YAP nuclear translocation.

Recently, the influences of extracellular microenvironment stiffness on chondrocyte intracellular Ca^2+^ signaling and its implications on OA progression have been explored. Transient receptor potential (TRP) channels are cation-selective transmembrane receptors, the activation of which cause an influx of cations, particularly Ca^2+^, leading to cytokine secretion and gene transcription to mediate cellular metabolism. Several members of TRP family exhibited extracellular stiffness-dependent activity. For example, in healthy chondrocytes, transient receptor potential vanilloid 4 (TRPV4) activity could be modulated by ECM viscoelasticity, resulting in low intracellular Ca^2+^ in fast-relaxation matrices and high intracellular Ca^2+^ in low-relaxation matrices [105]. This is attributed to dynamic cell volume change in response to matrix viscoelasticity. OA chondrocytes, however, lost their ability to do so, thus being trapped in a state with continuously high intracellular Ca^2+^ level, which caused inflammatory and catabolic gene expressions through Ca^2+^-dependent phosphorylation of glycogen synthase kinase 3β (GSK3β) [105].

By culturing chondrocytes in polydimethylsiloxane (PDMS) substrates with different stiffness, Du et al. discovered that intracellular Ca^2+^ influx was dominated by TRPV4 at higher stiffness (78-197Kpa), while piezo type mechanosensitive ion channel component 1/2 (PIEZO1/2) mainly mediated lower stiffness (2–54 kpa) substrate-induced Ca^2+^ response [106]. The selective activations of mechanosensors in response to variant microenvironment stiffness might explain the differences in metabolism of chondrocytes from normal or OA cartilage, which have distinct matrix stiffness.

Another member of TRP family, transient receptor potential ankyrin 1 (TRPA1), was reported to be overexpressed in OA chondrocytes. Activation of TRPA1 brought both positive and harmful effects on the chondrogenic phenotype of chondrocytes. The positive effects (synthesis of collagen II and IL-10) were strengthened while harmful effects (synthesis of collagen I and IL-6) were alleviated in softer matrix in contrast to stiffer matrix [96].

Overall, these Ca^2+^ channels acted as sensors of microenvironment stiffness to modulate the Ca^2+^ influx and intracellular Ca^2+^ dependent signaling. Extracellular matrix stiffness affects the activities of these channels through both direct and indirect manners [107]. Physical changes of plasma membrane in response to microenvironment stiffness, such as membrane tension and curvature, directly cause these channels to open. Indirect gating of these channels involves a multi-step process which starts with activations of mechanosensitive proteins (often G-protein coupled receptor) on the plasma membrane, followed by activations of intracellular cascades that lead to cytoskeleton reorganization, which finally regulate these channels opening [107].

It is well known that mechanical forces induce modulation of nuclear chromatin structures and epigenetically impact gene transcription [108]. The longevity protein α-Klotho plays a role in the attenuation of aging phenotype in tissues throughout the body. Iijima et al. uncovered that the loss of chondrocyte phenotype in age-related cartilage matrix stiffening was attributed to α-Klotho promoter methylation and decreased α-Klotho expression [97]. Mechanically, the effect of matrix stiffening on chondrocyte α-Klotho promoter methylation resulted from actin fiber formation and polymerization, and subsequent expression of DNA methyl transferase 1 (DNMT1), which is an enzyme that catalyzes the methylation and repression of gene transcription.

**Figure 2:**
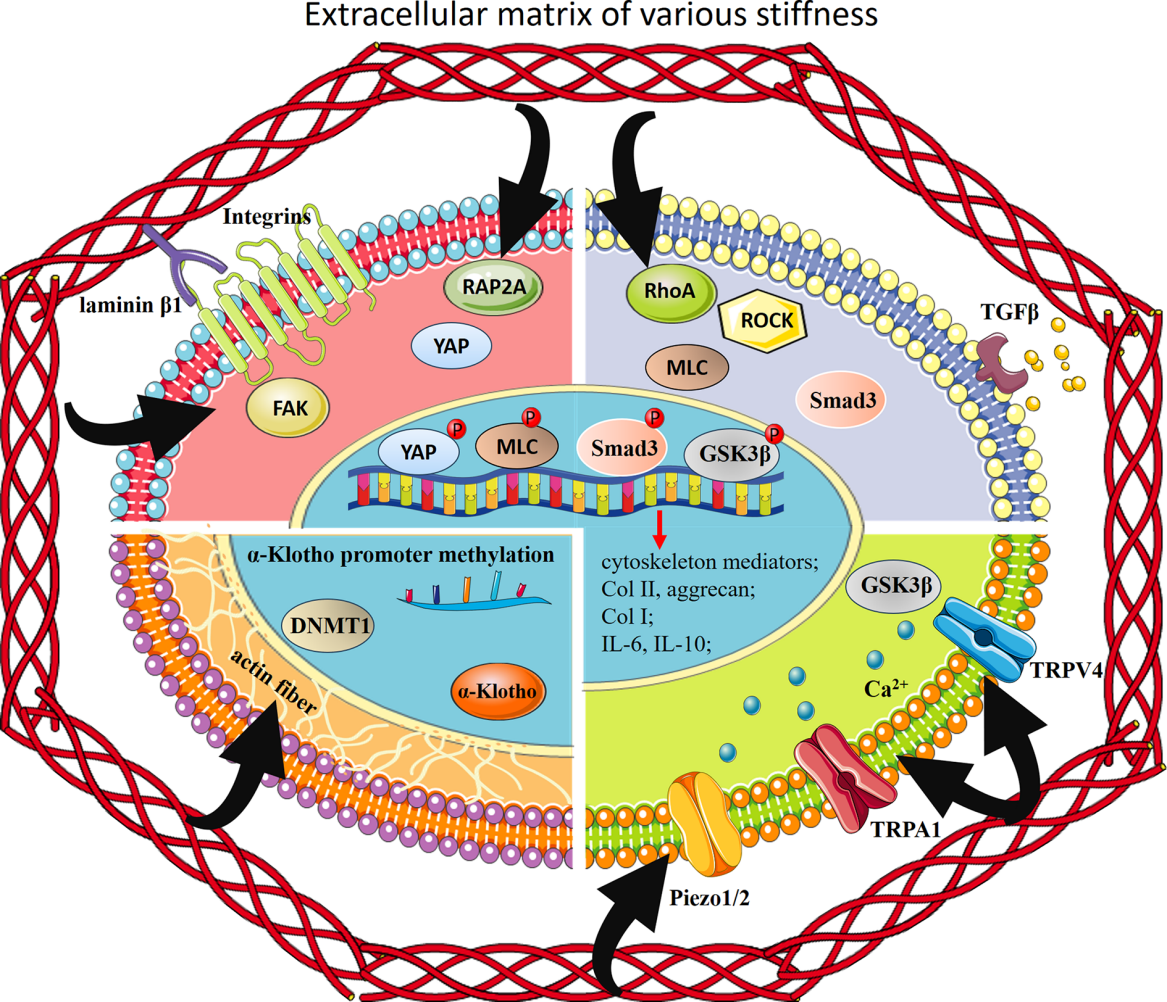
Mechanisms of matrix stiffness in regulating chondrocyte metabolism Scaffolds with distinct stiffness affected chondrocyte metabolism through mechanisms of either canonical mechano-transduction pathways such as laminin β1-FAK, RhoA-ROCK or Ca^2+^ channels TRPV4, TRPA1, Piezo1/2 and epigenetic modifications. Downstream events include phosphorylation of YAP, MLC, Smad3 and GSK3β, which activate genes involved in cytoskeleton reorganization, inflammation and metabolism of chondrocytes

Overall, scaffolds with distinct stiffness affected chondrocyte metabolism through mechanisms of either canonical mechano-transduction pathways such as laminin β1-FAK, RhoA-ROCK or Ca^2+^ channels TRPV4, TRPA1, PIEZO1/2 and epigenetic modifications. Downstream events include phosphorylation of YAP, MLC, SMAD3 and GSK3β, which activate genes involved in cytoskeleton reorganization, inflammation and metabolism of chondrocytes (Figure 2).

## Conclusions

Cartilage degradation and destruction have been considered as the major characteristics of OA. However, it is difficult to confirm these indicators at the early stage of OA because of the technique limitations. For decades, the cartilage matrix stiffness has become more and more attractive in OA diagnose, therapy and prognosis, since growing evidences suggested the robust correlation between altered cartilage stiffness and OA progression. However, because of the variances in cartilage sample collection and preparation, as well as in stiffness detecting and calculating methods, researchers have not reached an agreement on whether cartilage matrix stiffen or soften during cartilage degeneration in OA. Thus, future studies need to systematically elaborate how cartilage matrix stiffness altered in response to OA, with respect to the OA pathogeny, locations, and progressing stage.

Chondrocyte behaves as a viscoelastic solid, the stiffness of which is determined by its own cytoskeleton systems [45]. The expressions and organization patterns of cytoskeletal proteins change in response to extracellular matrix stiffness, thus making chondrocytes being able to sense the mechanical properties of microenvironment and adjust the stiffness of themselves to adapt to various microenvironmental stiffness [40]. Since cartilage is constantly bearing loading strains, the extent to which chondrocytes deform under these daily strains may play a role in the activation of mechanotransduction pathways, and may determine whether the cellular response is anabolic or catabolic. In other words, whether the strain loaded on cartilage is physiological or pathological is not only determined by the strain magnitude but also by how much strain is perceived and sensed by chondrocytes, which depends on both cartilage matrix stiffness and chondrocyte stiffness.

During OA progression, the alterations in cartilage matrix stiffness become a mechanical cue in breaking the balance between matrix protein synthesis and degradation, which finally feedback to affect the stiffness of cartilage matrix. Therefore, there is an interplay between cartilage matrix stiffness and chondrocyte metabolic activities. However, which one of these two critical events occurred earlier during OA initiation is still inconclusive. Based on the reports from lots of literatures, the etiology of OA (aging, obesity, inflammation, trauma and heredity) is a major factor in determining the initializing events that promote OA development. Another problem needed to be addressed in future is that whether alteration in cartilage matrix stiffness is beneficial or harmful for maintaining chondrogenic phenotype and metabolism during OA progression. Currently, almost all of the studies seemed to illustrate that physiological stiffness of cartilage matrix is beneficial for cartilage homeostasis, while any changes to matrix stiffness caused chondrocytes to lose their phenotype and shifted toward catabolic activities. Even though these evidences were convincing and consistent, we still need to keep in mind that matrix stiffness was not the only variant in these studies. Thus, it will be necessary to evaluate the effect of altered stiffness of cartilage matrix on chondrocytes and cartilage homeostasis based on more rigorous and meticulous experimental design, to exclude the interferences from other factors. A more comprehensive understanding about the positive or negative influences of matrix stiffness on cartilage degeneration and destruction will be helpful in developing scaffolds with optimal stiffness to achieve cartilage repair and regeneration concerning different stages, locations, and pathogenies of OA.

In conclusion, we systematically overviewed current understandings and debates regarding the correlation between OA development and cartilage stiffness, including the ECM, PCM and chondrocyte stiffness. The specific cartilage matrix molecules responsible for the pathological changes of cartilage stiffness during OA were also listed. Finally, targeting the stiffness of synthetic materials provides a novel strategy in the field of cartilage repair and regeneration after OA-induced cartilage damage. However, there were some significant limitations of this review. One of these is the lack of detailed elucidations of how the stiffness of cartilage matrix and chondrocytes were detected, such as the devices, the theory of different techniques, the formulations to calculate the modulus, etc. Another drawback is the failure to discriminate stiffness from other mechanical parameters of cartilage (equilibrium modulus, instantaneous modulus, aggregate modulus, shear modulus, Poisson’s ratio and viscoelasticity). Fortunately, these issues were clearly addressed and expatiated in recently published reviews [109,110].

## Data Availability

Data sharing is not applicable to this article as no datasets were generated or analysed during the current study.

## Abbreviations

ACLT: anterior cruciate ligament transection
ADAMT: a disintegrin and metalloproteinase with thrombospondin motifs
AFM: atomic force microscopy
AGE: advanced glycation end product
CHAD: chondroadherin
CS: chondroitin sulphate
Dcn: decorin
DMM: medial meniscus
ECM: extracellular matrix
FAK: focal adhesion kinase
GAG: glycosaminoglycan
GSK3β: glycogen synthase kinase 3β
HA: hyaluronic acid
HS: heparan sulfate
Hspg2: heparan sulfate proteoglycan2
ICRS: international Cartilage Repair Society
IL-10: interleukin-10
IL-1β: interleukin-1β
IL-6: interleukin-6
MACI: matrix-assisted autologous chondrocyte implantation
MIA: monosodium-iodoacetate
MLC: myosin light chain
MMP: matrix metalloproteinase
OA: osteoarthritis
OARSI: osteoarthritis Research Society International
PA: polyacrylamide
PCM: pericellular matrix
PDMS: polydimethysiloxane
PEGDMA: Poly ethylene glycol) dimethacrylate
PGs: proteoglycans
PTOA: post-traumatic osteoarthritis
RAP2A: ras-related protein 2a
RhoA: ras homolog gene family member A
ROCK: Rho-associated protein kinase
SLRP: small leucine rich proteoglycan
SNP: sodium nitroprusside
TGFβ: transforming growth factor β
TIMP: tissue inhibitor of metalloproteinase
TNF-α: tumor necrosis factor-α
TRPA1: transient receptor potential ankyrin 1
TRPV4: transient receptor potential vanilloid 4
YAP: yes-associated protein
